# Spatial Distribution and Flight Patterns of Two Grain Storage Insect Pests, *Rhyzopertha dominica* (Bostrichidae) and *Tribolium castaneum* (Tenebrionidae): Implications for Pest Management

**DOI:** 10.3390/insects11100715

**Published:** 2020-10-19

**Authors:** Joanne C. Holloway, Gregory J. Daglish, David G. Mayer

**Affiliations:** 1 New South Wales Department of Primary Industries, WWAI, Pine Gully Rd, Wagga Wagga NSW 2650, Australia; 2 Department of Agriculture and Fisheries, Dutton Park, Queensland 4102, Australia; Greg.Daglish@daf.qld.gov.au (G.J.D.); David.Mayer@daf.qld.gov.au (D.G.M.)

**Keywords:** stored grain, spatiotemporal patterns, ecology, pest management, flight activity

## Abstract

**Simple Summary:**

Lesser grain borer (LGB) and rust red flour beetle (RFB) are two common insect pests that cause severe economic damage to stored grain worldwide. Current treatments rely on chemicals, but both species have developed resistance to most of these. However, by understanding the ecology of these species in regional locations it is possible to develop more targeted pest management strategies. Therefore, we conducted a 2-year trapping study to investigate for the first time the spatial and temporal activity of these two species in a temperate region of southeastern Australia. Traps were located both on and off farms. Of the two species LGB were more common, and higher numbers of both species were found in traps close to grain storages. However, they both had a wide distribution as they were caught in all traps. Both species displayed distinct seasonal trends, with activity stopping over the colder, winter months in both years. The lack of activity is partly a response to the colder temperatures, with flight activity stopping below 14.5 °C for LGB and 15.6 °C for RFB. These results can be used to inform pest management activities such as cleaning of storages, monitoring for insects, resistance management, and site hygiene.

**Abstract:**

The lesser grain borer, *Rhyzopertha dominica*, and the rust red flour beetle, *Tribolium castaneum*, are two major beetle pests commonly found infesting stored products worldwide. Both species can cause severe economic damage and their management is complicated by their potential to develop resistance to several of the limited chemical options available. However, pest management strategies can be improved by understanding the ecology of the pest insect. To determine the spatiotemporal activity of *R. dominica* and *T. castaneum,* we conducted a trapping study over two years in a temperate region of south-eastern Australia, with traps located near grain storages and fields. We captured higher numbers of *R. dominica* than *T. castaneum*, and both species were more prevalent in traps located close to grain storages. Similar and consistent seasonal patterns were displayed by both species with activity ceasing during the winter (June–August) months. We found linear correlations between maximum daily temperatures and trap catches, and minimum threshold temperatures for flight activity were 14.5 °C and 15.6 °C for *R. dominica* and *T. castaneum*, respectively. The results are discussed in relation to the ecology of these pests along with their implications for pest management.

## 1. Introduction

Effective pest management is necessary for maintaining a high quality of stored grain. A large amount of information has been gathered on the mechanics of grain storage (e.g., aeration cooling and drying), as well as insecticide and fumigant treatments for pest control. However, the increase in the prevalence of resistance to the limited range of chemical treatments available [1,2,3,4,5,6] has resulted in a need for a more ecological approach to develop more targeted pest management strategies. 

The lesser grain borer, *Rhyzopertha dominica* (F.) (Coleoptera: Bostrichidae), and the red flour beetle, *Tribolium castaneum* (Herbst) (Coleoptera: Tenebrionidae), are two major beetle pests commonly found infesting stored grain worldwide. Both species can cause severe economic loss due to decreasing the quantity and quality of stored seeds, as well as the cost of treatment and preventative measures [7,8,9,10,11]. This is compounded by their potential to develop resistance to both fumigants [2,4,12,13] and grain protectant insecticides [3,6,14].

Ecological studies investigating spatiotemporal dynamics in the USA and Australia have shown that both *R. dominica* and *T. castaneum* can fly large distances, with dispersal flights of over 1 km reported as common [15,16,17,18,19]. Further, adults of both species disperse from existing infestations and invade clean resources [20,21]. This process is mediated by their responses to volatile chemicals [22,23]. Apart from stored grains, food resources for *R. dominica* include twigs and acorns [24,25], while *T. castaneum* can survive off certain fungi and decaying plant material [26].

Both species demonstrated a strongly seasonal pattern in flight activity in regions of North America that experience very cold winters [19,27,28,29,30,31]. The only published data on seasonal flight activity of *R. dominica* and *T. castaneum* in Australia come from subtropical regions [15,16,17]. While activity in these regions display some seasonality, flight activity continues throughout the year. However, most Australian cereal crops are grown in more southern temperate regions, where the seasonal range in temperature is much greater, and greater seasonality in flight activity might be more evident. In this paper, we examine this possibility using data from a trapping study conducted over two years in a temperate grain growing region in New South Wales. We discuss our results in relation to the ecology of these pests along with their implications for pest management. 

## 2. Materials and Methods

All trapping was conducted in the farming district around the town of Wagga Wagga (Latitude: −35.108169°, Longitude: 147.359832°), in the Riverina district of southern New South Wales. Farms in this region are primarily mixed enterprises, comprising winter crops, and sheep and cattle grazing. Canola and cereal grains, such as wheat, barley and oats are typically sown in autumn/winter (April–June) and harvested in spring/summer (November–January). The traps were located on seven properties (five farms, a commercial seed company, and a research farming station) all within a 45 km arc to the north and east of Wagga Wagga. All properties had multiple silos, and were mixed farming properties with native trees and vegetation surrounding the paddocks. Wheat and canola were the dominant crops in the landscape during the trapping period, and most silos contained wheat seed. 

Traps consisted of Lindgren four-funnel traps (Contech Inc., Delta, BC, Canada), with propylene glycol as the preserving agent in the collecting cup. Four traps were placed on each property: two traps were located near the grain silos (‘silo traps’) and two located several hundred metres from the silos near the edge of a cropping paddock (‘field traps’). Median distance between pairs of silo and field traps was 17.4 m (range 10.1–47.0 m) and 38.6 m (range 30.9–51.7 m), respectively. The median distance between the silo and field traps was 0.28 km (range 0.18–1.18 km). The trap locations were chosen to provide maximum distance between the silo and field traps, while allowing unimpeded access with minimal disturbance to the property owners for the duration of the study. The traps were suspended so that the collection cups were approximately 1.5 m from the ground. One silo trap and one field trap at each property was baited with a rice weevil, *Sitophilus oryzae* (L.) aggregation pheromone lure (Insect Limited Inc., Westfield, IN, USA) from January 2014 to May 2015, and from May 2015 to June 2016 these traps held a rusty grain beetle, *Cryptolestes ferrugineus* (Stephens, 1831), aggregation pheromone lure (Research Directions Pty Ltd., Brisbane, Queensland, Australia). No *S. oryzae* were captured and the *C. ferrugineus* results have been published elsewhere [32]. This paper is based on analysis of *R. dominica* and *T. castaneum* caught in these traps.

All collection cups and lures on the traps were replaced every four weeks, apart from one occasion in December 2014 when the traps were serviced after only 2 weeks. Data from this shorter trapping period were not analysed. Insects from the collection cups were transferred to the laboratory at Wagga Wagga Agricultural Institute where they were placed in ethyl alcohol until they could be identified. Daily minimum and maximum ambient temperature records were downloaded for the nearest official regional weather station located at Wagga Wagga airport (latitude −35.165278°, longitude 147.466389°) [33].

Trap counts were analysed with Poisson generalised linear mixed models (GLMMs) using restricted maximum likelihood (REML) in GenStat [34]. The over-dispersed Poisson distribution with the log link function was adopted for the data. The fixed effects were location, year, season, and their interactions, with an offset of the number of days that the traps were in operation. The seasons were winter (June–August), spring (September–November), summer (December–February) and autumn (March–May). The random effects were farms and traps and were restricted so negative estimated variance components were not permitted. Type III testing (backwards elimination of terms from the full model) was used to select parsimonious models—location interactions, and the offset of days, were all non-significant and so were omitted in the final models. 

The relationship between trap catch and temperature was analysed using a threshold (nonlinear bent-stick) model [34]. This model estimates a temperature below which no activity occurs, with a linear increase in numbers after this threshold. Therefore, mean daily maximum temperatures were calculated across each sample period and analysed against the total number of *R. dominica* or *T. castaneum* trapped during that period.

## 3. Results

Our results were based on the by-catch from a trial to determine flight activity of *S. oryzae* and *C. ferrugineus* [32]. Aggregation pheromones are highly species-specific and Stevens [35] found a lack of attraction exerted by either of these lures on both *R. dominica* and *T. castaneum*. Further, when analysed we found no significant effect on trap catch due to the presence or absence of the *S. oryzae* or *C. ferrugineus* lures (*R. dominica*: F_1,128_ = 2.25, *p* = 0.136; *T. castaneum*: F_1,128_ = 0.01, *p* = 0.943). Therefore, the presence or absence of these lures was not considered in any model and the two traps near grain storage and the two field traps at each property were pooled for the statistical analyses.

**Figure 1:**
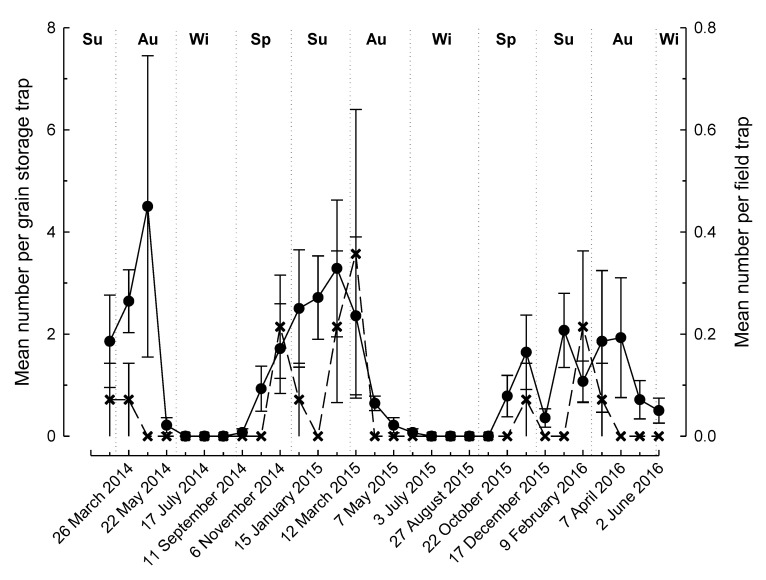
Mean number (±SE) of *Rhyzopertha dominica* caught in Lindgren funnel traps located near grain storages (•) or near fields (**×**) in the Riverina district, Australia (Seasons of the year (Su = summer, Au = autumn, Wi = winter, Sp = spring) are indicated at the top of the figure).

**Figure 2:**
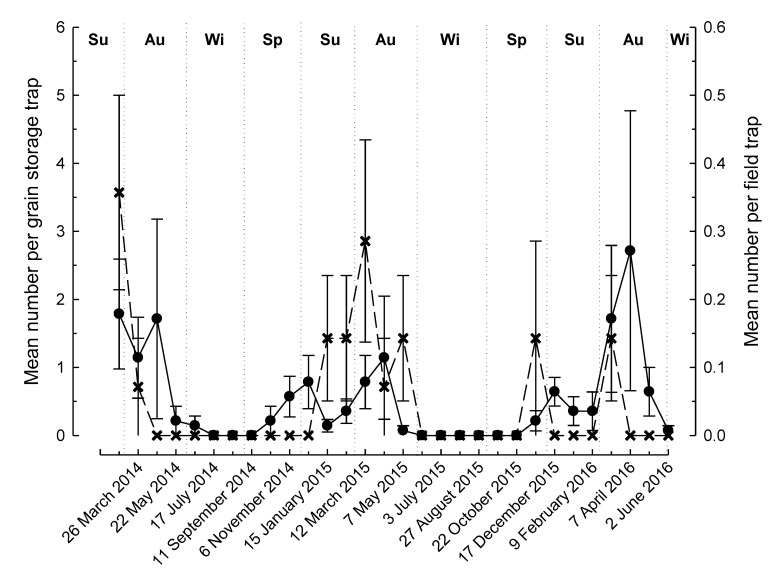
Mean number (±SE) of *Tribolium castaneum* caught in Lindgren funnel traps located near grain storages (•) or near fields (**×**) in the Riverina district, Australia (Seasons of the year (Su = summer, Au = autumn, Wi = winter, Sp = spring) are indicated at the top of the figure).

There were significant differences in the number of beetles captured, with over twice as many *R. dominica* than *T. castaneum* (504 and 241, respectively) caught in the traps over the 122-week study period (F_1,119_, *p* = 0.014). The location of the traps (near grain storage vs. field) was found to significantly affect the trap catch for both *R. dominica* (F_1,128_ = 36.34, *p* < 0.001, Figure 1) and *T. castaneum* (F_1,128_ = 26.08, *p* < 0.001, Figure 2). We caught over 25 times more *R. dominica* (485 near grain storage cf. 19 in field traps) in traps over the course of the study near grain storages than those in the fields (Figure 1), whereas we captured approximately 10 times more *T. castaneum* (220 near grain storage cf. 21 in field traps) (Figure 2). Both species were trapped at all sites, but the number caught was variable ranging from a total of 21–174 and 1–6 for *R. dominica* traps near grain storages and in fields, respectively, and 3–91 near grain storages and 0–8 in fields for *T. castaneum*. 

For both species, we found a significant seasonal effect on trap captures (*R. dominica*: F_3,128_ = 2.76, *p* = 0.045; *T. castaneum*: F_3,128_ = 5.20, *p* = 0.002). Flight activity displayed relatively similar temporal patterns both near grain storages and in the field for both species (Figure 1 and Figure 2). Peak activity mostly corresponded with the summer months (December–February), while a cessation of activity occurred during the winters (June–August) of both years (Figure 1 and Figure 2).

**Figure 3:**
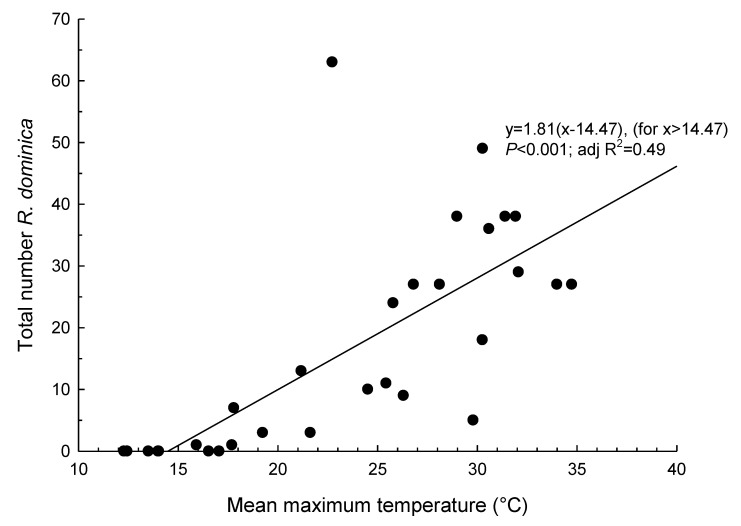
Relationship between mean maximum temperature during sampling period and total number of *Rhyzopertha dominica* caught in Lindgren funnel traps in the Riverina district, Australia.

**Figure 4:**
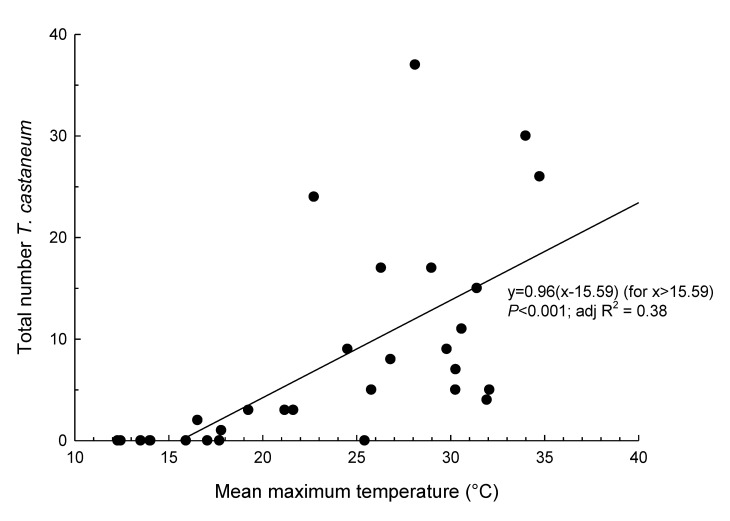
Relationship between mean maximum temperature during sampling period and total number of *Tribolium castaneum* caught in Lindgren funnel traps in the Riverina district, Australia.

With the reduction of flight activity coinciding with the cooler months, activity in both species appears to be related to temperature. Using the analysis of the number of beetles caught in traps and mean maximum temperatures observed during the sampling periods, we found significant bent-stick linear relationships in both *R. dominica* (Figure 3) and *T. castaneum* (Figure 4). Based on these analyses, we found that flight activity did not commence until the mean maximum daily temperature exceeded 14.47 ± 2.99 °C for *R. dominica* and 15.59 ± 3.30 °C for *T. castaneum*. Above these temperatures, there were significant positive relationships between mean maximum temperature and trap catches. Mean maximum temperature explained 49% and 38% of the variation in *R. dominica* and *T. castaneum* trap numbers, respectively. 

## 4. Discussion

We found that the spatiotemporal activity patterns for *R. dominica* and *T. castaneum* captured in south-eastern Australia over the two years of our study were very similar. Both species displayed consistent strong seasonal trends, with cessation of activity during winter months. In addition, activity was greater around grain storages, with higher numbers of both species captured in traps located close to grain storages than those near fields. These results replicate those found for *C. ferrugineus* trapped in the same region [32]. This spatial distribution is probably due to the storage areas providing food resources in the form of stored grain and residues.

Seasonal activity patterns similar to those in our study were found for both *R. dominica* and *T. castaneum* throughout North American regions where winter temperatures often fall below freezing [28,29,30,31,36]. However, in milder regions, such as South Carolina, low level activity may persist throughout the year [37]. Flight activity in Australia appears to be the same. Our study, located in a southern temperate region, displayed a distinct seasonal pattern, whereas in those conducted in subtropical southern and central Queensland the pattern was less evident [15,16,17,38]. Similar geographic patterns were found in India [39].

Regional differences in temperature may be partially responsible for the disparity in seasonal activity patterns. In our study, mean daily maximum temperatures only explained 49% and 38% of the variation in *R. dominica* and *T. castaneum* trap numbers, respectively. This is consistent with other trapping studies [28,32,40,41,42]. Consequently, other factors have been proposed, such as density, age, cropping habitat, landscape, light intensity, and starvation level, which may exert additional influences on the flight activity in these beetles [18,21,22,43,44,45,46]. 

Using a laboratory flight chamber, Dowdy [44] determined the lower threshold for flight initiation was 19.9 °C in *R. dominica*. Additionally, using flight chambers, Cox et al. [47] found a similar minimum temperature for *R. dominica*, and that of 25 °C for *T. castaneum*. However, based on Australian field conditions, Wright and Morton [48] estimated this temperature was 16 °C for *R. dominica* when calculated using mean hourly temperatures and trap captures. This is comparable with our results using mean maximum temperatures. Further, trap captures of *T. castaneum* in rice mills in Arkansas, USA, indicate that this species is active when mean daily temperatures are only around 10–15 °C [49]. These studies highlight the importance of investigating the ecology of a pest within its natural habitat, particularly as the results can be used to determine effective pest management strategies.

Several studies worldwide have trapped both *R. dominica* and *T. castaneum* away from grain storage facilities [15,16,17,19,27,28,40,50,51]. While the prevalence of the beetles is generally higher closer to the grain storage, the degree of spread may differ between species. Similar to our study, Daglish et al. [15] reported *R. dominica* was more widespread in trap captures than *T. castaneum* in central Queensland, Australia. However, the distribution was more uniform, with equal numbers of *R. dominica* captured in traps located in and away from grain depots. In the USA it has been reported that *R. dominica* consume several non-cereal seeds [24,25], while *T. castaneum* are attracted to specific fungi [23,26]. However, native food sources in Australia remain unknown. These could assist in explaining the differences in their spatial distributions.

Genetic analysis of both *R. dominica* and *T. castaneum* in regional Queensland found that the beetles were genetically homogenous, despite the widespread nature of the captures [16,17]. Molecular studies of *C. ferrugineus* have shown there to be one contiguous population throughout the whole of eastern Australia [52], and we infer that it is possible that a similar scenario exists for *R. dominica* and *T. castaneum*. 

Implications for pest management: Understanding the ecology of insect pests of stored grain can assist in their management. The seasonal patterns of flight activity of *R. dominica* and *T. castaneum* in this region of Australia have been revealed for the first time and will allow pest managers of stored grain to understand when their sites are most vulnerable to attack, as well as prime times to clean and treat storage structures.

Based on our results, we recommend that storage structures and machinery should be emptied, cleaned and treated during the winter months (June–August) when the threat of reinfestation from flying beetles is at its lowest. Monitoring of grain in storage via direct sampling in the grain mass should be maintained throughout the year but be most vigilant during the summer months when dispersal activity is at its peak. This strategy is especially important given that *R. dominica*, a primary pest of grain that causes severe economic damage to stored products [24], is the most widespread and prevalent species. Further, based on field studies the majority of female *R. dominica* and *T. castaneum* have mated prior to dispersal [16,17,53], thus providing a mechanism for a rapid increase in the establishing population.

The large dispersal range has implications for the spread of genes inferring resistance to the limited range of pesticides available. In particular, *R. dominica* is a highly adaptive species that has developed resistance to several stored grain insecticides [2,3,54]. This resistance may be exacerbated by the high degree of polyandry found in both *R. dominica* and *T. castaneum* captured in flight [53]. Multiple matings increase the probability of an individual female carrying genes for resistance. Consequently, the chances of introducing resistant progeny to newly infested sites are increased.

Finally, given the high degree of dispersal throughout a region, we emphasise the importance placed on hygiene at storage facilities. Stored grain insects respond to volatiles released by food sources such as wheat, although these may differ between species [55]. Further, the attraction of these volatiles can be influenced by the presence of insects in the substance [22]. Consequently, we feel that it is important that grain spills are promptly removed and destroyed, particularly during those times of peak flight activity.

## 5. Conclusions

While greater numbers of *R. dominica* were caught than *T. castaneum*, spatiotemporal activity in the temperate region of south-eastern Australia was similar in both species and consistent over the two-year study. Both *R. dominica* and *T. castaneum* were more prevalent in traps located close to grain storage facilities than those in the field. However, both species were caught at all trap locations indicating a large geographical distribution. Activity was correlated with temperature and ceased during the winter months with minimum flight threshold temperatures of 14.5 °C and 15.6 °C for *R. dominica* and *T. castaneum*, respectively. These consistent patterns have implications for pest management and can lead to greater efficacy in the strategies employed.
